# Complete Stress–Strain Relations of Early-Aged Cementitious Grout under Compression: Experimental Study and Constitutive Model

**DOI:** 10.3390/ma15031238

**Published:** 2022-02-07

**Authors:** Gang Peng, Xiaopeng Hu, Ditao Niu, Shuai Zhong

**Affiliations:** 1 School of Civil Engineering, Xi’an University of Architecture and Technology, Xi’an 710055, China; xauat_pg0331@163.com (G.P.); shs339@sina.com (X.H.); zhongshuai@xauat.edu.cn (S.Z.); 2 State Key Laboratory of Green Building in Western China, Xi’an University of Architecture and Technology, Xi’an 710055, China

**Keywords:** cementitious grout, energy evolution principle, damage variable, stress–strain constitutive model

## Abstract

The compressive stress–strain behaviors of early-aged cementitious grout specimens were experimentally investigated, and the differences of characteristic parameters of the stress–strain curve and the energy evolution law of each specimen under uniaxial compression were discussed in this study. The results indicate that with an increase in the specimen age, the peak stress, peak strain, ultimate strain, elastic modulus, peak secant modulus, strain ductility coefficient, and energy-dissipation coefficient of the prism specimens gradually improved. Additionally, a comparison of the test results of cementitious grout specimens and concrete specimens with the same age reveals that the peak stress, peak strain, and ultimate strain of cementitious grout specimens were greater than that of concrete specimens, the elastic modulus and peak secant modulus of the specimens were less than that of concrete specimens, and the strain ductility coefficient and energy-dissipation coefficient show no consistent conclusions with respect to the material type. Moreover, comparing the energy evolution curves of specimens with different specimen ages shows that the decrease rate of the elastic strain rate and the increase rate of the dissipated energy rate gradually decreased with the increase in specimen age. The elastic strain rate and dissipated energy rate of the CGM−270 specimen and control specimens were greater than that of other specimens, and the decrease rate of the elastic strain rate was less than that of other specimens. Based on the statistical damage theory, a statistically stochastic damage constitutive model was derived by considering the characteristics of cementitious grout according to the compression test results. A comparison of the proposed models with the experimental results indicated that the proposed stress–strain constitutive models were sufficiently accurate.

## 1. Introduction

A cementitious grouting material has the characteristics of good fluidity (vibration-free), micro expansion, the rapid development of early strength (the 3-day compressive strength is larger than 40 MPa), and high strength (greater than 60 MPa) after mixing with water in a certain proportion, and have been widely used in the reconstruction and reinforcement of concrete structures as a strengthening material [1,2]. Owing to their widespread application, cementitious grouts have attracted research attention [3,4,5,6,7,8,9]. However, the current research on cementitious grouts is focused mainly on the optimization of materials and the mechanical properties of cementitious grouts, and research on the stress–strain behavior is relatively limited [10,11,12,13,14,15]. In addition, for reinforced concrete structural members strengthened with cementitious grout, investigation of the stress–strain behavior of the cementitious grout and the development of stress–strain constitutive models are necessary for bearing capacity analysis. Moreover, as a material with rapid development of early strength, a good understanding of the mechanical properties and stress–strain behaviors of early-aged cementitious grouts is conducive to shortening the wet operation time of cementitious grouts in practical engineering applications and accelerating the construction period. Hence, it is necessary to study the compressive stress–strain behaviors of early-aged cementitious grouts.

In this study, four types of cementitious grout specimens and concrete specimens (control group) were adopted, the compressive stress–strain constitutive relationships were investigated by considering the different ages of the specimens (1, 2, 3, 7, and 28 days), and the test results of cementitious grout specimens were compared with that of the control specimens. Moreover, the energy evolution regulations of specimens with different testing ages and types of cementitious grouts were explored and discussed. According to the test results, compressive stress–strain constitutive models for the four types of cementitious grouts under uniaxial compression were proposed with consideration of the specimen age. The research results—particularly the proposed stress–strain constitutive model—provide a theoretical basis for bearing-capacity analysis of reinforced concrete structural members strengthened with cementitious grouts.

## 2. Experimental Program

### 2.1. Materials

**Figure 1:**
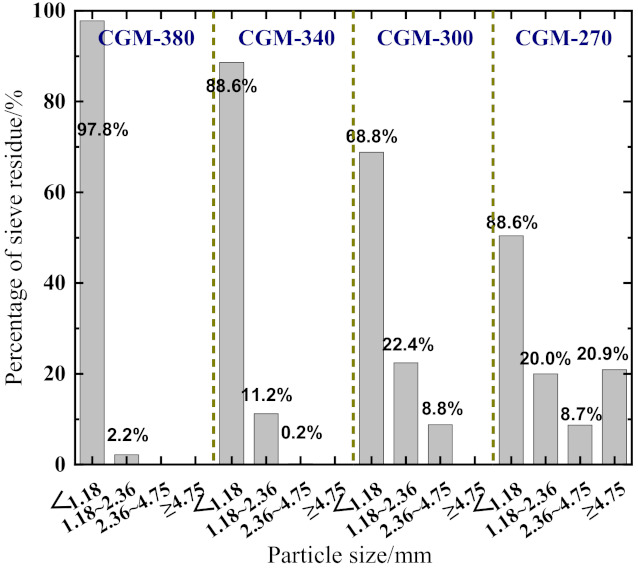
Results of sieving analysis test.

Four types of cementitious grouts, i.e., CGM−380, CGM−340, CGM−300, and CGM−270, which were produced by Beijing Sino–sina Building Technology Co., Ltd., Beijing, China, were used in this study. A sieving analysis test was conducted, and the results are presented in Figure 1. As shown, except for CGM−270, the maximum particle sizes of the other three types of cementitious grouts were <4.75 mm. Tap water was used for mixing, and the mass ratios of water to CGM were 0.32, 0.16, 0.125, and 0.095 for CGM−380, CGM−340, CGM−300, and CGM−270, respectively. After the cementitious grouts were directly mixed with water and poured into a mold, cementitious grout samples with different dimensions were obtained. The specimens were cured in standard conditions (a temperature of 20 ± 3 °C and a relative humidity greater than 90%) for 1 day, after which the molds were stripped off and then specimens were cured under natural environmental conditions for more than 28 days. The main performance indices of the cementitious grouts, such as the fluidity, vertical expansion rate, and compressive strength, were measured to ensure that the cementitious grouts used in this study conformed to the specifications of the technical code for the application of cementitious grout (GB/T 50448-2015) [16,17]. The test results are presented in Table 1.

Concrete with a cube compressive strength close to that of cementitious grouts was used as the control specimens; the mix proportions of the concrete are shown in Table 2. Ordinary Portland cement (P.O 42.5) was adopted as the cementing material in this study. Medium sand with fineness modulus >2.85, and crushed stones with an aggregate size ranging from 5 to 25 mm were used as fine aggregate and coarse aggregate, respectively. The crushing value index and apparent density of the crushed stones were about 1.3% and 2.7 g/cm^3^, respectively. An RD-N polycarboxylate superplasticizer was used for the fabrication of the specimens, and the addition amount was approximately 1–2% of the mass of the cementitious materials. The specimens were cured under the same conditions as the cementitious grout specimens.

### 2.2. Testing Methods

**Figure 2:**
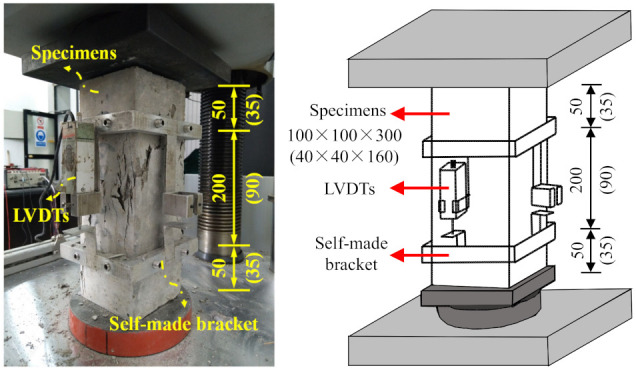
Diagram of test equipment (unit: mm).

In accordance with GB/T 50448–2015 [16] and GB/T 50081-2019 [18], for the axial compression test, three prism specimens with dimensions of 40 × 40 × 160 mm^3^ were fabricated for CGM−380, CGM−340, and CGM−300, respectively, and three specimens with dimensions of 100 × 100 × 300 mm^3^ were fabricated for CGM−270 and the control specimens, respectively. A 3000-kN universal hydraulic testing machine was used to obtain a stable and complete compressive stress–strain curve. Linear variable differential transformers were fixed on both sides of the specimens with an aluminum bracket (see Figure 2); the compression deformation values were measured within a gauge distance of 200 mm on both sides of the 100 × 100 × 300 mm^3^ specimens and within a gauge distance of 90 mm on both sides of the 40 × 40 × 160 mm^3^ specimens. The displacement control mode was adopted, with a slip rate of 0.2 mm/min. Before the formal test, to reduce the effects of the uneven surfaces of the specimens and the gaps between the specimens and the upper and lower pressure-plate surfaces, the specimens were preloaded via the following steps: they were loaded to approximately 30% of the axial compressive strength and then unloaded; this was repeated thrice. The compression deformation and load were recorded using a TDS-602 dynamic data acquisition system, which produced by Tokyo Measuring Instrument Research Institute, Tokyo, Japan. The loading device used for the uniaxial compression test of the prism specimens is shown in Figure 2.

## 3. Test Results and Discussion

### 3.1. Cubic Compressive Strength

**Figure 3:**
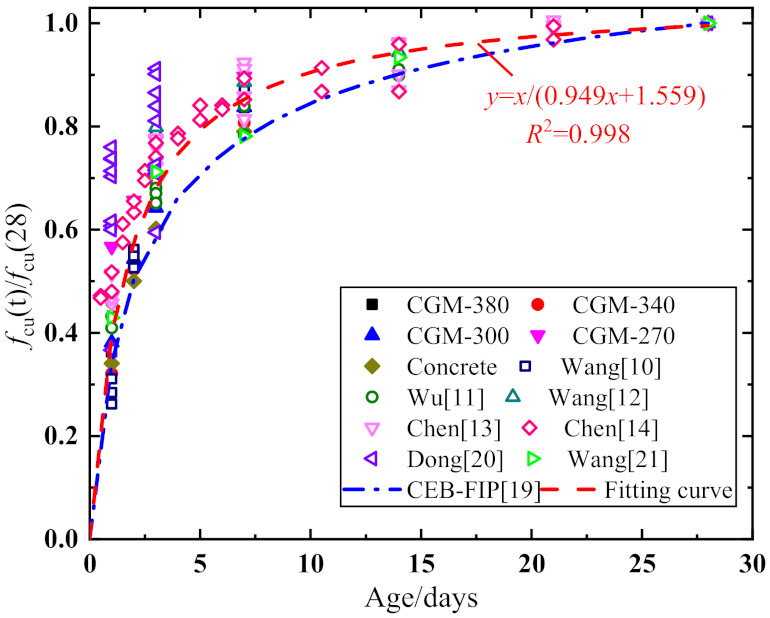
Variations of cubic compressive strength with respect to testing age.

Figure 3 displays the normalized cubic compressive strength of the early-aged cementitious grout. As shown, for the cementitious grouts and control specimens, the strength increased rapidly in the first 7 days; then, the increase gradually slowed, and the strength tended to be stable after 7 days. Comparisons between the cementitious grouts and the control specimens show that the cementitious grouts, especially the CGM−270 specimens, had the obvious characteristics of rapid development of early strength. Particularly, the 1-day compressive strength and 3-day compressive strength were 34.1 and 60.2% of the 28-day compressive strength for control specimens, and the 1-day compressive strength and 3-day compressive strength can reach 56.7 and 77.4% of the 28-day compressive strength for CGM−270 specimens. Although there are some differences among the experimental cube compressive strength of the four groups of cementitious grout specimens, the results were described by the same mathematical model for the convenience of application. The test data were regressed with reference to CEB-FIP [19], and the formula for calculating the cubic compressive strength of the cementitious grouts was obtained, as shown in Equation (1). The experimental results from different sources are also shown in Figure 3 for comparison [10,11,12,13,14,20,21]. The calculated results of the proposed cubic compressive strength calculation model agreed well with the results reported by other scholars, which indicates that the proposed model can be used to calculate the cube compressive strength of early-aged cementitious grouts:

(1) $$f_{\mathrm{cu}}(t)=f_{\mathrm{cu}}(28)\cdot \left(\frac{t}{1.559+0.949t}\right)$$

where, $f_{\mathrm{cu}}(t)$ and $f_{\mathrm{cu}}(28)$ are the cube compressive strength of the cementitious grout at testing ages *t* (1 ≤ *t* ≤ 28) and 28 days, respectively. 

### 3.2. Uniaxial Compression Test Results

#### 3.2.1. Failure Modes

**Figure 4:**
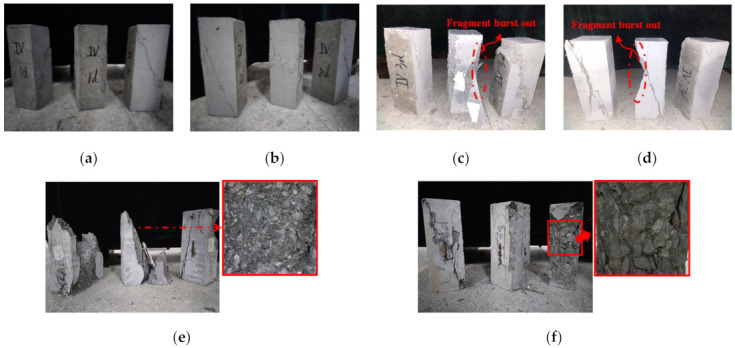
Typical failure modes of (**a**) Ⅳ-1 d; (**b**) Ⅳ-2 d; (**c**) Ⅳ-3 d; (**d**) Ⅳ-7 d; (**e**) Ⅳ-28 d; and (**f**) C-28 d specimens.

The failure process of cementitious grout and control prism specimens under uniaxial compression can be divided into four stages: elastic deformation, stable crack development, unstable crack development, and descending. For the specimens with ages of 1, 2, and 3 days, cracks appear at the edges and corners of the specimen first, and the crack width increases gradually and extends up and down with the increasing loading. The specimen is damaged after the edge and corner cracks are penetrated, with the failure process showing certain plastic failure characteristics. For the specimens with ages of 3 days and above, vertical cracks appear in the middle of the prism specimen first, and then the cracks continue to extend and develop; the width of the cracks increase, and the main cracks gradually penetrate with the loading increase. There are fragments bursting out during the failure with a large crack sound, showing that the failure process presents obvious brittle failure characteristics. Comparing the failure characteristics of the prism specimens of cementitious grouts with the control specimens reveals that the cementitious grout specimens, especially the CGM−380, CGM−340, and CGM−300 specimens, had a shorter crack development stage. These specimens are more prone to crack and their bearing capacity is rapidly lost after reaching the peak stress. The integrity of the specimen is poor after failure, as shown in Figure 4. Moreover, compared with the failure surface of the specimen, it can be found that when the cementitious grout specimens are damaged, the aggregate passing through the failure surface is split, and there is no cohesive failure between the aggregate and the cement paste grouting gel. The failure surface is smooth, which is significantly different from that of the control specimens.

#### 3.2.2. Stress–Strain Curves

**Figure 5:**
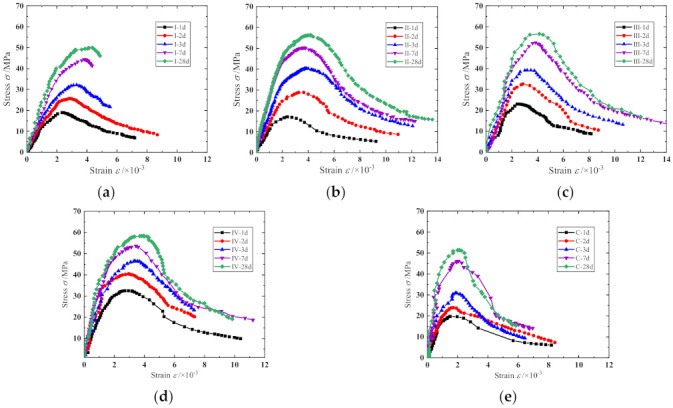
Typical compressive stress–strain curves of (**a**) CGM−380; (**b**) CGM−340; (**c**) CGM−300; (**d**) CGM−270; and (**e**) concrete specimens.

The complete uniaxial stress–strain curves of specimens are presented in Figure 5. As shown, except for cementitious grout specimens Ⅰ-3 d, Ⅰ-7 d, and Ⅰ-28 d, all the uniaxial stress–strain curves are composed of complete ascending and descending branches. The stress–strain curve is close to a straight line at the beginning of loading. As the loading continues, the strain increase accelerates, and the stress gradually increases to the peak point. The stress at the peak point is considered as the axial compressive strength for the prismatic specimens. In the descending branch of the curve, the curve bends downward first and gradually changes in the concave direction, and an inflection point is generated. The stress–strain curve gradually protrudes to the strain axis once the inflection point is passed, and it gradually exhibits a convergence trend.

**Figure 6:**
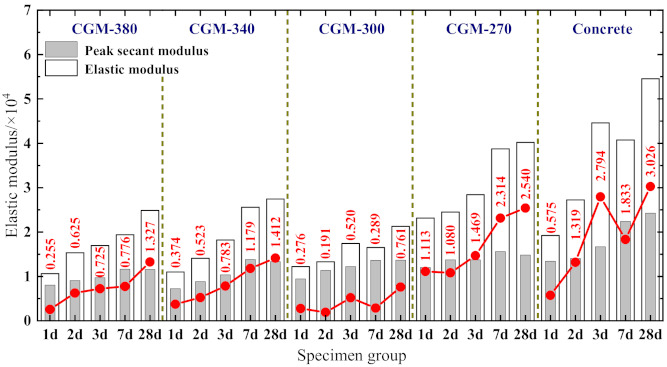
Discrepancies between peak secant modulus and elastic modulus.

**Figure 7:**
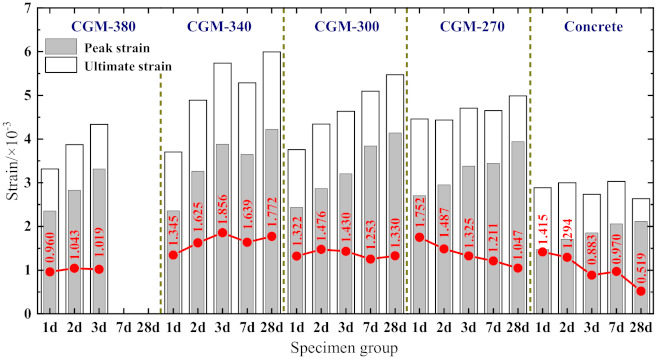
Discrepancies between ultimate strain and peak strain.

It can be observed from Figure 5 that there are obvious differences between the stress–strain curves of specimens of different ages. For the ascending branch of the stress–strain curves, the slope increased in varying degrees, and the linear elastic deformation stage of the curves extended with the increase in specimen age, indicating the elastic modulus of the specimens increased gradually. Moreover, the non-linear elastic deformation stage of the curves also extended with the increasing specimen age, which shows that the peak strain, peak secant modulus, and the difference between the peak secant modulus and the elastic modulus increased gradually (see Figure 6). As for the post-peak branch, the curve became steeper gradually with the increase in specimen age, indicating that the specimen gradually shows significant brittle characteristics, which is consistent with the conclusion, shown in Figure 7, that the difference between the ultimate strain (the strain at a stress equal to 85% of the peak stress in the descending branch, *ε*_0.85_) and the peak strain of the specimen generally shows a decreasing trend. A comparison of the stress–strain curves of the prism specimens with the same age indicates that the peak stress of specimens generally shows as CGM−270 > CGM−300 > CGM−340 > CGM−380 ≈ concrete specimens. In addition, comparisons also show that the peak strain and ultimate strain of cementitious grout specimens were greater than those of concrete specimens, while the elastic modulus and peak secant modulus were smaller than those of concrete specimens, which indicate that the uniaxial compression stress–strain curve of concrete specimens had a steeper ascending branch. In addition, as Figure 7 shows, the discrepancies between ultimate strain and peak strain of the CGM-380 specimens were less than that of the other four groups, which is consistent with the fact that the stress–strain curves of the CGM−380 specimens had a steeper post-peak branch.

#### 3.2.3. Characteristic Parameters of Stress–Strain Curves

1. Peak stress *σ*_1_

**Figure 8:**
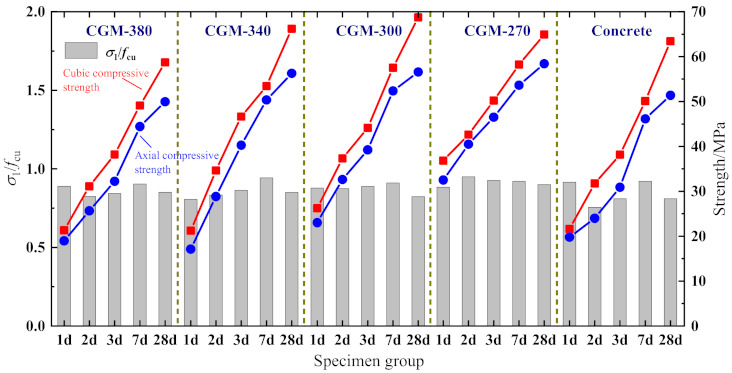
Ratio of axial compressive strength to cubic compressive strength.

**Figure 9:**
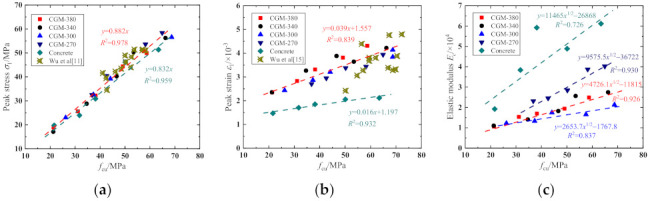
Relationship between (**a**) peak stress; (**b**) peak strain; and (**c**) elastic modulus of compressive stress–strain curve and cubic compressive strength.

The ratios of axial compressive strength to cubic compressive strength are presented in Figure 8. As shown, the variations of the axial compressive strength of each specimen group with age are basically consistent with that of the cubic compressive strength. The strength ratios of CGM−380, CGM−340, CGM−300, and CGM−270 are 0.824−0.904, 0.806−0.943, 0.823−0.910, and 0.900−0.950, respectively, which are slightly greater than those of concrete specimens (about 0.756−0.921). Compared with the concrete specimens, the curve of cubic compressive strength and axial compressive strength of cementitious grout has a larger slope in the first 3 days, which is consistent with the characteristics of the rapid development of early strength of cementitious grout. Figure 9a displays relationship between peak stress and the cubic compressive strength. According to the linear trend of strength shown in Figure 9a, combined with the provisions of GB 50010-2010 [22], regression analysis was conducted for all compressive strength test data obtained from cementitious grout specimens and the following unified strength conversion relationship was obtained:

(2) $$\sigma_{1}=0.882f_{cu}$$

The correlation coefficient *R*^2^ of the above regression formula is 0.978 (see Figure 9a). Based on the test data, the conversion coefficient between the axial compressive strength and the cubic compressive strength of cementitious grout is 0.882, which is greater than that of the concrete specimens (about 0.832), but it is still in the range of 0.7−0.92 [23]. In order to verify the rationality of the proposed strength conversion model, the test results from Wu’s work [11] are given in Figure 9a. Comparisons show that the proposed strength conversion relationship is in good agreement with the test data of other scholars.

2. Peak strain *ε*_1_

Figure 9b presents the variations in the peak strain with respect to the cubic compressive strength. As shown, the peak strain of cementitious grout specimens was obviously greater than that of concrete specimens, and thus, the existing formula for calculating the peak strain of concrete was not suitable for cementitious grout specimens. In addition, Figure 9b also shows that the peak strain increased linearly with an increase in the cubic compressive strength. According to the regression analysis of the test data in [23,24], the following formula for calculating the peak strain of cementitious grout specimens was obtained:

(3) $$\varepsilon_{1}=3.94\times 10^{-5}f_{cu}+1.557\times 10^{-3}$$

The correlation coefficient *R*^2^ of the above regression formula was 0.839. Comparing the calculated results with the test data in Wu et al. [15], it can be seen that the test data in Wu et al. [15] are relatively discrete, but basically distributed on both sides of the fitting curve, and the change trend of the test data agrees well with the formula curve, indicating that the proposed formula can be used to calculate the peak strain of the cementitious grout specimens.

3. Elastic modulus *E_c_*

The elastic modulus is defined as the secant modulus of the ascending section of the stress–strain curve from the origin to the point corresponding to 40% of the peak stress [25]. Figure 9c displays the relationship between elastic modulus and cubic compressive strength. It can be observed that the elastic modulus of the early-aged specimens increased with the age. Take the CGM−380 specimens as an example, the elastic modulus of CGM−380 specimens increased from 1.061 × 10^4^ to 2.486 × 10^4^ MPa with the specimen age increase from 1 d to 28 d. In addition, comparing the elastic modulus for the specimens with the same ages reveals that the elastic modulus of control specimens was far greater than that of cementitious grout specimens, which is consistent with the conclusions given by Wu et al. [11,15]. Moreover, comparisons also show that the CGM−270 specimen had the larger elastic modulus than the other three types of cementitious grouts. This is because the elastic modulus of the specimen was greatly affected by the elastic modulus of the aggregate. The elastic modulus of the coarse aggregate in the concrete specimen and CGM−270 specimen was relatively large, which results in the relatively large elastic modulus of the specimen as a whole. Based on the relationship between the elastic modulus and cubic compressive strength given in [26], the regression analysis was conducted on test data according to Equation (4), and the results of the regression analysis are listed in Figure 9c.

(4) $$E_{c}=af_{cu}^{1/2}+b$$

4. Strain ductility coefficient *β*

**Figure 10:**
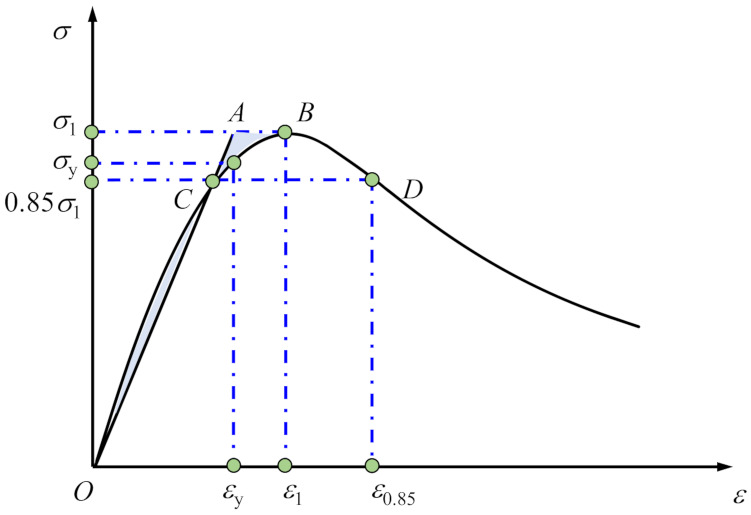
Diagram showing the calculation of strain ductility coefficient.

To quantitatively analyze the mechanical and deformation properties of the cementitious grout, the ratio of the ultimate strain to the initial yield strain was defined as the strain ductility coefficient of the early-aged cementitious grout specimens, as indicated by Equation (5). The initial yield point can be determined via the energy equivalence method [27]. As shown in Figure 10, make a secant OC through the origin of the coordinate axis so that the area enclosed by OC and the skeleton curve is equal to the area of ABC, then the vertical projection of point A on the skeleton curve is the initial yield point, and its corresponding stress and strain are the initial yield stress and initial yield strain of the specimen, respectively.

(5) $$\beta =\frac{\varepsilon_{0.85}}{\varepsilon_{y}}$$

Here, *β* represents the strain ductility coefficient of the early-aged cementitious grout specimen, and *ε*_0.85_ and *ε_y_* represent the ultimate strain and initial yield strain, respectively, of the specimen. 

**Figure 11:**
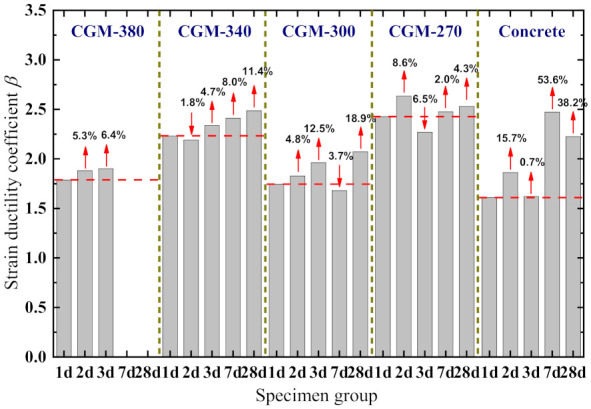
Strain ductility coefficient of specimen.

The strain ductility coefficients of each of the specimens are displayed in Figure 11. In the figure, there are no values for the ductility coefficients of the CGM−380 specimens with testing ages of 7 and 28 days owing to the lack of an effective post-peak section. As shown in Figure 11, the strain ductility coefficients show no consistent variations with the increase in specimen age but generally had a positive correlation with the specimen age for some specimen groups. Further, it can be seen that for the specimens with ages of 3 days or less, the strain ductility coefficients of cementitious grout specimens were greater than that of the control specimens, which is closely related to the characteristics of the rapid development of early strength of cementitious grout. For the specimens with ages of 3 days or more, however, there were no consistent variations of the strain ductility coefficient for different groups of specimens, which generally shows as CGM−270 ≈ control specimens ≈ CGM−340 > CGM−380 ≈ CGM−300. 

#### 3.2.4. Analysis of Energy Evolution Law

The deformation and failure process of cementitious grout specimens under compression is the process of internal energy evolution and exchange with the outside [27]. With the continuous action of external compressive stress, the energy input from the outside is gradually transformed into the elastic strain energy and dissipation energy of the specimen, which can be expressed as [28]:

(6) $$U=U_{e}+U_{d}$$

(7) $$U=\int \sigma d\varepsilon =\sum_{i=1}^{n}\frac{1}{2}(\sigma_{i}+\sigma_{i-1})(\varepsilon_{i}-\sigma_{i-1})$$

(8) $$U_{e}=\frac{\sigma_{i}^{2}}{2E}$$

(9) $$U_{d}=\sum_{i=1}^{n}\frac{1}{2}(\sigma_{i}+\sigma_{i-1})(\varepsilon_{i}-\sigma_{i-1})-\frac{\sigma_{i}^{2}}{2E}$$

where, U is the total strain energy of specimens, which is the energy input by the external compression pressure, and can be calculated by Equation (7); $U_{e}$ is the elastic strain energy of specimens, which can be obtained from Equation (8); and $U_{d}$ is the dissipative energy of specimens, which can be obtained based on the total strain energy and elastic strain energy of specimens, see Equation (9). $\sigma_{i}$ and $\sigma_{i-1}$ are the axial stress obtained from the *i* th and (*i* − 1) th data acquisition, respectively. $\varepsilon_{i}$ and $\varepsilon_{i-1}$ are the strain obtained from the the *i* th and (*i* − 1) th data acquisition, respectively. *n* represents the number of data acquisitions. For comparative analysis, the stress and strain data before the ultimate strain $\varepsilon_{0.85}$ are taken to calculate the total strain energy *U.*

1. Energy-dissipation coefficient *η_e_*

**Figure 12:**
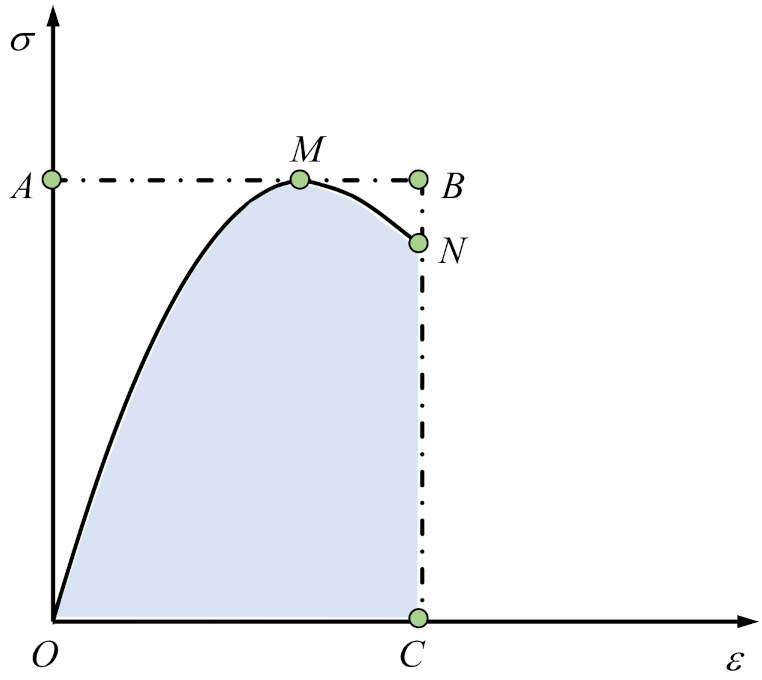
Diagram for calculation of energy-dissipation coefficient.

The energy-dissipation coefficient is introduced to study the energy dissipation of specimens in this study. The diagram for calculation of the energy dissipation coefficients of the specimens is shown in Figure 12, and the formula for calculation of the energy-dissipation coefficient is as follows [29]:

(10) $$\eta_{e}=\frac{S_{0\mathrm{MNC}}}{S_{0\mathrm{ABC}}}$$

where *η_e_* is the energy- dissipation coefficient of the early-aged cementitious grout specimen, and *S*_0MNC_ represents the area enclosed by the stress–strain curve between 0.85 times the peak stress in the descending branch and the coordinate axis, i.e., the total strain energy of specimens. *S*_0ABC_ represents the area of the rectangle enclosed by the strain value corresponding to 0.85 times the peak stress at the descending branch, the peak stress, and the coordinate axis.

**Figure 13:**
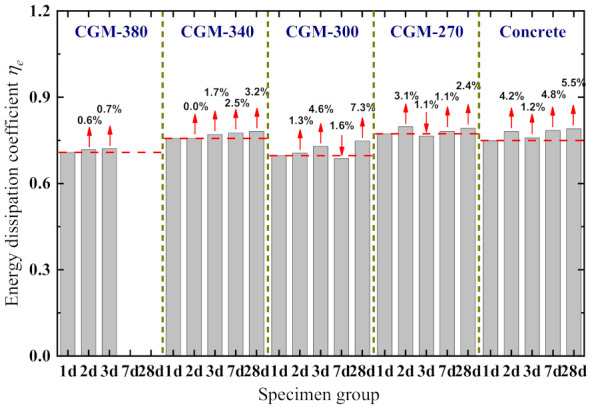
Energy-dissipation coefficient of specimen.

The energy-dissipation coefficients of each specimen are presented in Figure 13. As shown, with the increase in specimen age, the energy-dissipation coefficients of each specimen show an overall increasing trend, but the variations are small (the maximum increases are 0.7, 3.2, 7.3, 3.1, and 5.5%, respectively). Comparing the energy-dissipation coefficients of specimens with the same age reveals that the energy-dissipation coefficients of specimens fabricated with different materials do not exhibit any significant variation, but on the whole, the energy-dissipation coefficients of CGM−270 and control specimens are greater than those of other specimens. Specifically, the energy-dissipation coefficients of CGM−380 specimens vary from 0.708 to 0.721, with an average value of 0.716 and a standard deviation of 0.007. In addition, the energy-dissipation coefficients of CGM−270 specimens vary from 0.773 to 0.797, with an average value of 0.781 and a standard deviation of 0.013.

2. Energy evolution law

**Figure 14:**
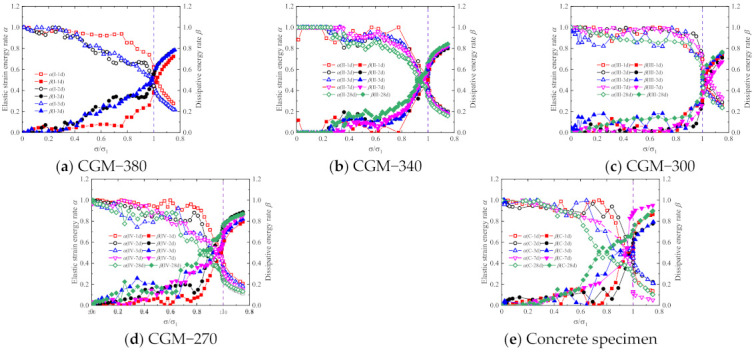
Variations of energy evolution of (**a**) CGM−380; (**b**) CGM−340; (**c**) CGM−300; (**d**) CGM−270; and (**e**) concrete specimens.

**Figure 15:**
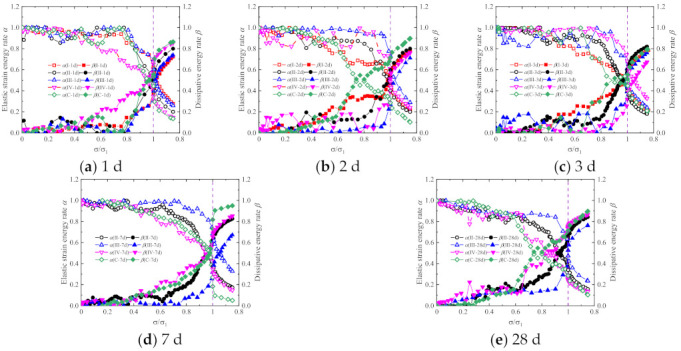
Variations of energy evolution of (**a**) 1 day; (**b**) 2 days; (**c**) 3 days; (**d**) 7 days; and (**e**) 28 days specimens.

The elastic strain energy rate $\alpha =U_{e}/U$ and dissipation energy rate $\beta =U_{d}/U$ are defined to represent the energy dissipation in the loading process. The variations of elastic strain energy rate α and dissipation energy rate β with normalized stress $\sigma /\sigma_{1}$ are presented in Figure 14 and Figure 15. For the same reason, no results were obtained for the energy-dissipation coefficients of the CGM−380 specimens with testing ages of 7 and 28 days.

As shown in Figure 14 and Figure 15, the elastic strain energy rate and dissipation energy rate show similar variations for the specimens with different testing ages and types of cementitious grouts. Generally, with the increasing loading, the elastic strain energy rate decreased while the dissipation energy rate increased. Specifically, when $\sigma_{i}/\sigma_{1}<0.7$, the specimen is in the stage of internal crack closure. The closure of internal cracks leads to an increase in dissipated energy, and finally results in the gradual decrease in the elastic strain rate and the gradual increase in the dissipated energy rate. When $0.7<\sigma_{i}/\sigma_{1}<1$ the specimen is in the stage of rapid propagation of internal cracks. At this time, the internal cracks of the specimen expand rapidly, the micro defects gradually evolve into macro cracks, the elastic strain rate decreases rapidly, and the dissipation energy rate increases rapidly. With the continuous loading, the specimens enter the post-peak stage, and the elastic strain of the specimens begins to release. The crack propagation and friction between crack surfaces lead to the continuous increase in the dissipated energy; the elastic strain rate continues to decrease and the dissipated energy rate continues to increase. As Figure 14 shows, there are no obvious differences in the energy evolution law of specimens with different ages, but on the whole, with the increase in age, the decrease rate of the elastic strain rate and the increase rate of the dissipated energy rate gradually decrease. This is because the longer the curing age, the greater the slurry strength and the fewer micro defects in the specimen, the more energy needs to be consumed for the evolution of micro defects in the process of load. Figure 15 shows that the elastic strain rate and dissipated energy rate of the CGM−270 specimen and the control specimens are close, and on the whole, the increase rate of the dissipated energy rate is greater than that of other specimens, and the decrease rate of the elastic strain rate is less than that of other specimens. This may be because there are more interfaces between the coarse aggregate and slurry in the CGM−270 specimen and control specimens, and the interface dislocation and friction consume more energy in the process of loading.

## 4. Stress–Strain Constitutive Relationships

Based on the theory of damage mechanics, under the action of compression load, the damage in the prismatic specimen of the cementitious grout gradually accumulates, the effective bearing area in the specimen gradually decreases, and the nominal stress and effective stress of the specimen meet the following relationship [30,31]:

(11) σ=F/A

(12) $$\sigma^{*}=F/A^{*}$$

(13) $$\sigma =\frac{A^{*}}{A}\sigma^{*}$$

where, *σ* and *σ*^∗^ represents the nominal stress and effective stress of the specimen, respectively, MPa; *F* represents the compression load acting on the specimens, N; and *A* and *A*^∗^ represent the nominal bearing area and effective bearing area of the specimen respectively, mm^2^.

The damage variable is used to characterize the development of internal damage, which is usually defined as the ratio of the internal damage area of the specimen to the nominal bearing area [32,33], as follows:

(14) $$D=\frac{A-A^{*}}{A}=1-\frac{A^{*}}{A}$$

where, *D* represents damage variable of the specimens, 0 ≤ *D* ≤ 1; *D* = 0 means that there is no damage inside the specimens, and *D* = 1 means that the specimen is completely damaged.

The following relationship can be obtained from Equations (13) and (14):

(15) $$\sigma =\left(1-D\right)\sigma^{*}$$

According to the strain coordination principle, the strain of the damaged part of the specimen is consistent with that of the undamaged part [34]. Assuming that the undamaged part in the specimen meets the law of elastic deformation, Equation (15) can be transformed into:

(16) $$\sigma =\left(1-D\right)E\varepsilon$$

### 4.1. Ascending Branches

When the strain is less than the peak strain of the specimen (*ε* ≤ *ε*_1_), the relationship between the damage variable *D* and the specimen strain *ε* can be described by the Weibull distribution [35], as follows:

(17) $$\frac{dD}{d\varepsilon }=\frac{p}{q}{\left(\frac{\varepsilon }{q}\right)}^{p-1}\exp\left[-{\left(\frac{\varepsilon }{q}\right)}^{p}\right]$$

where, *p*, *q* are the shape parameter and scale parameter of the Weibull distribution, and both are greater than 0. The integral is taken on both sides of Equation (17), so:

(18) $$D=1-\exp\left[-{\left(\frac{\varepsilon }{q}\right)}^{p}\right]$$

The following formula can be obtained from Equations (16) and (18):

(19) $$\sigma =E\varepsilon \exp\left[-{\left(\frac{\varepsilon }{q}\right)}^{p}\right],\varepsilon \leq \varepsilon_{1}$$

According to the following geometric conditions on the ascending branches of compression stress–strain curves: (1) *ε* = 0, *σ* = 0; (2) *ε* = 0, d*σ*/d*ε* = *E*; (3) *ε* = *ε*_1_, *σ* = *σ*_1_; and (4) *ε* = *ε*_1_, d*σ*/d*ε* = 0, the expressions of parameters *p* and *q* can be obtained as follows [35]:

(20) $$p=\frac{1}{\ln\left(E_{c}\varepsilon_{1}/\sigma_{1}\right)},q=\frac{\varepsilon_{1}}{{\left(1/p\right)}^{1/p}}$$

### 4.2. Descending Branches

When the strain is greater than the peak strain of the specimen (*ε* ≥ *ε*_1_), the lognormal distribution can be used to describe the compression stress–strain relationship of the specimens [36].

(21) $$\sigma =a\exp\left[-0.5{\left(\frac{\ln\left(\varepsilon /b\right)}{c}\right)}^{2}\right]$$

The parameters *a*, *b* in Equation (21) are the peak stress *σ*_1_ and peak strain *ε*_1_ of the specimen, respectively, so Equation (21) can be transformed into:

(22) $$\sigma =\sigma_{1}\exp\left[-0.5{\left(\frac{\ln\left(\varepsilon /\varepsilon_{1}\right)}{c}\right)}^{2}\right],\varepsilon \geq \varepsilon_{1}$$

The final expression of the statistical damage constitutive model of the specimen can be obtained from Equations (19) and (22), as follows:

(23) $$\sigma =\left\{\begin{array}{ll} E\varepsilon \exp\left[-{\left(\frac{\varepsilon }{q}\right)}^{p}\right] & \varepsilon <\varepsilon_{1}\\ \sigma_{1}\exp\left[-0.5{\left(\frac{\ln\left(\varepsilon /\varepsilon_{1}\right)}{c}\right)}^{2}\right] & \varepsilon \geq \varepsilon_{1} \end{array}\right.$$

According to Equation (16), the damage variable of specimens can be expressed as:

(24) $$D=\left\{\begin{array}{ll} 1-\exp\left[-{\left(\frac{\varepsilon }{q}\right)}^{p}\right] & \varepsilon <\varepsilon_{1}\\ 1-\frac{\sigma_{1}}{E\varepsilon }\exp\left[-0.5{\left(\frac{\ln\left(\varepsilon /\varepsilon_{1}\right)}{c}\right)}^{2}\right] & \varepsilon \geq \varepsilon_{1} \end{array}\right.$$

where, the parameters *p*, *q* and *c* can be obtained by regression analysis of the specimen data in the ascending and descending branches of the compression stress–strain curves, respectively. Then, the variations of parameters *p*, *q*, and *c* with age *t* (0 < *t* ≤ 28 days) can be given in the form of a logarithmic function with unknown parameters *m_i_*, *n_i_* (*i* = 1, 2, 3), as Equation (16) shown, and the unknown parameters *m_i_*, *n_i_* (*i* = 1, 2, 3) can be finally obtained by regression analysis, see Table 3.

(25) $$\left\{\begin{array}{c} p=m_{1}\ln(t)+n_{1}\\ q=m_{2}\ln(t)+n_{2}\\ c=m_{3}\ln(t)+n_{3} \end{array}\right.$$

**Figure 16:**
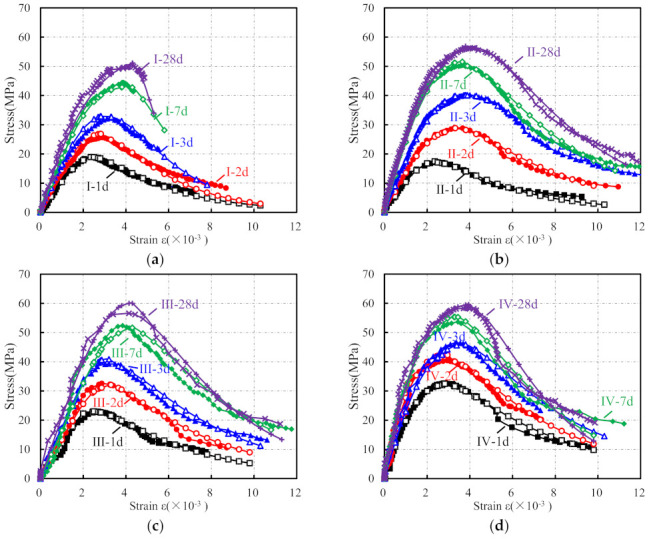
Comparisons between calculated curves from the proposed model and test results of (**a**) CGM−380; (**b**) CGM−340; (**c**) CGM−300; and (**d**) CGM−270.

Based on the statistical damage constitutive model and the calculation model of elastic modulus, peak stress, and peak strain proposed above, the complete stress–strain curves of early-aged cementitious grout under compression can be calculated. The comparisons between the calculation curves and the test results are shown in Figure 16. It can be seen that, although there are some discrepancies between the proposed models and test results, an acceptable level of agreement was observed in their comparisons, which indicates that the statistical damage constitutive model proposed in this paper can effectively characterize the compressive stress–strain relationship of early-aged cementitious grout. 

**Figure 17:**
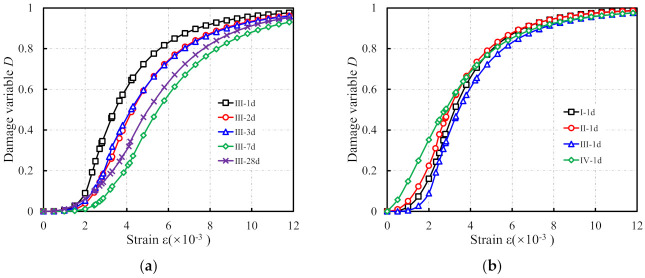
Variations of damage variable *D* of (**a**) CGM−300 specimens; and (**b**) 1 d specimens.

The CGM−300 group of specimens can be taken as an example to compare the differences between damage variables of specimens with different ages, see Figure 17a. As shown, the damage variable *D* in the model can well predict the damage evolution process of early-aged cementitious grout. In addition, it can also be seen from Figure 17a that the damage variable of the specimen under the same strain is larger with the increase in specimen age. This is because the cement slurry with increased strength can better inhibit the generation, extension, and penetration of internal cracks, and reduce the damage development rate of specimens. As Figure 17b shows, 1 day specimens are taken as an example to analyze the damage variables of specimens of different materials at the same age. It can be seen from Figure 17b that there is little difference in the damage evolution of specimens of different materials at the same age. Moreover, under the same strain, the IV-1 d specimen has a greater damage variable, which may be because the coarse aggregate contained in the CGM−270 cementitious grout increases the interface in the specimen, resulting in the relatively rapid development of damage of CGM−270 cementitious grout. 

## 5. Conclusions

The main objective of this study was to investigate the stress–strain behaviors of early-aged cementitious grouts. According to the test results and discussions presented herein, the following conclusions are drawn.

1. The prism specimen of cementitious grout experienced four main stages under the uniaxial pressure: elastic deformation, stable crack development, unstable crack development, and descending, and the deformation characteristics of each group are different at each stage. The brittleness characteristics of the specimens were more obvious with the increasing age. For the specimens with ages of 3 d or more, there are fragments breaking out when the specimens are damaged, accompanied by a large cracking sound. Compared with the control specimens, the cementitious grout specimens had relatively short crack-development stages, and had poor integrity after the failure. Moreover, the failure surface of the cementitious grout specimens was flat and smooth.
2. With an increase in the specimen age, the peak stress, peak strain, ultimate strain, elastic modulus, and peak secant modulus, along with the strain ductility coefficient and energy-dissipation coefficient increased to varying degrees, the brittleness of the specimens became obvious. Comparing the uniaxial compression characteristics of specimens with the same age reveals that the peak stress, peak strain, and ultimate strain of the cementitious grout specimens were greater than that of the concrete specimens; the elastic modulus and peak secant modulus of the specimens were less than that of concrete specimens; and the strain ductility coefficient and energy-dissipation coefficient shows no consistent conclusions with respect to the material type.
3. The results of the energy evolution analysis show that the elastic strain energy rate decreased while the dissipation energy rate increased with the increasing loading. Moreover, with the increase in age, the decrease rate of the elastic strain rate and the increase rate of the dissipated energy rate gradually decrease. Comparing the energy evolution curves of specimens with different types of cementitious grouts shows that the elastic strain rate and dissipated energy rate of the CGM-270 specimen and the control specimens are greater than that of other specimens, and the decrease rate of the elastic strain rate are less than that of other specimens.
4. Considering the effects of specimen age on the peak stress, peak strain, and elastic modulus, the calculation models of the peak stress, peak strain, and elastic modulus were established based on the experimental data. Moreover, based on the statistical damage theory, together with the characteristics of cementitious grouts, a statistically stochastic damage constitutive model suitable for early-aged cementitious grouts was established. Good agreement was observed between the calculated curves and the test results, indicating that the proposed stress–strain curve calculation model can accurately describe the deformation characteristics of early-aged cementitious grouts under uniaxial compression.

## Figures and Tables

**Table 1:** Test results of fluidity, vertical expansion rate, and compressive strength of cementitious grouts.

Indexes			CGM−380	CGM−340	CGM−300	CGM−270
Flow cone fluidity/s	Initial values	Limit values	≤35	-	-	-
		Test results	33.57	-	-	-
	30 min	Limit values	≤50	-	-	-
		Test results	49.89	-	-	-
Truncated cone fluidity/mm	Initial values	Limit values	-	≥340	≥290	≥650
		Test results	-	383	342	802
	30 min	Limit values	-	≥310	≥260	≥550
		Test results	-	356	294	582
Vertical expansion rate/%	3 h	Limit values	0.1~3.5			
		Test results	0.13	0.41	0.15	0.16
	Discrepancies between 24 h and 3 h	Limit values	0.02~0.50			
		Test results	0.02	0.16	0.08	0.08
Compressive strength/MPa	1 day	Limit values	≥15	≥20		
		Test results	18.9	26.4	27.9	39.7
	3 days	Limit values	≥30	≥40		
		Test results	34.7	42.8	48.6	47.1
	28 days	Limit values	≥50	≥60		
		Test results	59.3	65.7	69.1	62.6

**Table 2:** Mix proportions of the concrete.

Ingredient/kg∙m^−3^					28 d Compressive Strength/MPa
Cement	Sand	Crushed Stone	Water	Superplasticizer	
500	648	1152	150	2.50	63.4

**Table 3:** Regression results of coefficients *m_i_* and *n_i_* (*i* = 1, 2, 3).

Material Type	*p*		*q*		*c*	
	*m*_1_	*n*_1_	*m*_2_	*n*_2_	*m*_3_	*n*_3_
CGM−380	−0.535	2.877	0.603	3.668	−0.146	0.722
CGM−340	−0.304	2.299	0.475	4.028	−0.040	0.761
CGM−300	−0.599	4.972	0.775	3.530	−0.066	0.811
CGM−270	−0.136	1.441	0.138	3.569	−0.092	0.831

## Data Availability

Data is contained within the article.
